# Environmental Contaminants Exposure and Preterm Birth: A Systematic Review

**DOI:** 10.3390/toxics7010011

**Published:** 2019-03-01

**Authors:** Maria Grazia Porpora, Ilaria Piacenti, Sara Scaramuzzino, Luisa Masciullo, Francesco Rech, Pierluigi Benedetti Panici

**Affiliations:** Department of Maternal and Child Health and Urology, University of Rome “Sapienza”, Policlinico “Umberto I”, 00161 Rome, Italy; mariagrazia.porpora@uniroma1.it (M.G.P.); ilaria.piacenti@uniroma1.it (I.P.); luisa.masciullo@uniroma1.it (L.M.) francesco.rech@uniroma1.it (F.R.) pierluigi.benedettipanici@uniroma1.it (P.B.P.)

**Keywords:** air pollution, environmental toxicants, environmental exposure, particulate matter, drinking water contaminant, tobacco smoke, polycyclic aromatic hydrocarbons, pregnancy outcomes, preterm birth, obstetrical complications

## Abstract

Preterm birth is an obstetric condition associated with a high risk of infant mortality and morbidities in both the neonatal period and later in life, which has also a significant public health impact because it carries an important societal economic burden. As in many cases the etiology is unknown, it is important to identify environmental factors that may be involved in the occurrence of this condition. In this review, we report all the studies published in PubMed and Scopus databases from January 1992 to January 2019, accessible as full-text articles, written in English, including clinical studies, original studies, and reviews. We excluded articles not written in English, duplicates, considering inappropriate populations and/or exposures or irrelevant outcomes and patients with known risk factors for preterm birth (PTB). The aim of this article is to identify and summarize the studies that examine environmental toxicants exposure associated with preterm birth. This knowledge will strengthen the possibility to develop strategies to reduce the exposure to these toxicants and apply clinical measures for preterm birth prevention.

## 1. Introduction

Preterm birth (PTB) is defined as a delivery that occurs before 37 weeks of pregnancy. It can be one of the most important causes of mortality and morbidity in newborns, particularly in the case of very early PTB occurring before 32 weeks of pregnancy. Many studies have shown that PTB can be associated with several problems in lung and neurological development [1,2,3,4]. PTB represents a worldwide health problem. Unfortunately, its etiology is multifactorial and, in most cases, remains poorly understood. PTB risk factors are classified as modifiable factors (anemia, smoking, physical activity, prenatal care, nutrition, infections, obesity, and exposure to pollutants) and non-modifiable factors like phenotypic and genotypic ones.

PTB is caused by several factors such as: demographic factors, genetic predisposition, nutritional status, pregnancy history, and comorbidity (diabetes mellitus and hypertension/preeclampsia), infections, cervical dysfunction, placental abruption, uterine overdistention, fetal intrauterine growth restriction (polyhydramnios or multifetal pregnancy) [5]. In this wide range of risk factors, exposure to environmental pollutants during pregnancy has received increasing interest from researchers, and many studies have analyzed the relationship between environmental contaminants and the risk of PTB [6]. In fact, environmental pollutants (pesticides, polycyclic aromatic hydrocarbons, particulate matter, and toxic metals) could influence the pathogenesis of PTB [5,7].

This systematic review will identify and summarize the published studies on the possible role of environmental toxicants exposure in PTB. This knowledge will strengthen the possibility to identify pollutants that may be involved in PTB etiology in order to elaborate strategies to reduce such exposure and implement clinical measures for PTB prevention.

## 2. Materials and Methods

### 2.1. Search Strategy 

We conducted a literature search in PubMed and Scopus databases identifying articles published from 1 January 1992 to January 2019 on the exposure to toxicants and the occurrence of PTB. We used any combination of the following key words: air pollution OR environmental toxicants OR environmental exposure OR particulate matter OR drinking water contaminant OR persistent organic pollutants OR non-persistent chemicals OR polycyclic aromatic hydrocarbons OR toxic metals OR tobacco smoke OR electronic cigarettes OR global warming AND pregnancy outcomes OR preterm birth OR obstetrical complications OR birth weight.

### 2.2. Inclusion Criteria

Studies were included if they described exposure to environmental toxic compounds in the mother or gestational compartment (e.g., amniotic fluid, umbilical cord blood, or placental tissue) during pregnancy and if they analyzed differences in these levels among preterm delivery subjects compared to term delivery subjects. We considered accessible full-text articles written in English, clinical studies, original studies, and reviews. We examined the evidence of the association between environmental contaminants and preterm birth across the studies and we grouped them by type of contaminant (air pollutants, organic pollutants, toxic metals, tobacco, e-cigarette, and global warming).

This protocol was developed according to the reporting guidelines established by the Preferred Reporting Items for Systematic reviews and Meta-Analyses (PRISMA) 2009.

### 2.3. Exclusion Criteria 

The studies were excluded if they were: not written in English, OR duplicates OR irrelevant population OR Irrelevant exposures OR irrelevant outcomes OR patients with known risk factors for PTB.

Therefore, from 351 records identified through database synthesis, we included 78 studies in the qualitative synthesis.

## 3. Results

### 3.1. Environmental Compounds

#### 3.1.1. Air Pollutants

Air pollution represents one of the most important environmental risk factors for human health; the only way to avoid related clinical effects is to reduce the levels of exposure. The negative effects on health comprise a wide range of clinical outcomes, from general discomfort (burning eyes, allergies) to hospitalization for respiratory diseases, lung cancer, and death [8,9,10].

Air pollution can be generally defined as the presence of a complex mixture of gases (e.g., carbon monoxide [CO], nitrogen monoxide [NO], nitrogen dioxide [NO_2_], sulphur dioxide [SO_2_], ozone [O_3_], particles [solid or liquid]) which are released directly from various sources or formed by the interaction of the primary pollutants in the atmosphere [11].

The relationship between air pollution and obstetric complications has received great attention; in fact, epidemiological evidence has shown significant association between air pollution exposure during pregnancy and adverse gestational outcomes, such as low birth weight (LBW), intrauterine growth restriction (IUGR), and PTB [12,13,14]. Moreover, preterm newborns could have severe respiratory, cardiovascular, and neurodevelopmental disorders [15]. The physiopathological base of these associations is not fully understood, but inflammation and oxidative stress seem to play an important role [16]. Air pollutants could alter alveolar cell differentiation and maturation leading to neonatal respiratory distress syndrome and reactive airway disease [17]. During pregnancy, the oxygen demand is higher, the ventilation rate increases, and a faster fat accumulation process could lead to the accumulation of pollutants. In addition, as a result of toxics competition with hemoglobin binding sites, O_2_ availability to the fetus can be reduced, negatively affecting their development [17].

##### Particulate Matter (PM)

PM is composed of several chemical species, classified according to their aerodynamic diameter in PM10 (mass concentration in the atmosphere of particles with an aerodynamic diameter < 10 μm) and PM2.5 (aerodynamic diameter < 2.5 μm). Their composition and size are strongly related to their toxicity: the smaller the particles, the major their toxic effects because of their capacity to penetrate deep in regions of the lung and to translocate to the systemic circulation [18]. Exposure during pregnancy is associated with elevated blood pressure and high risk of pre-eclampsia [19]. A meta-analysis conducted by Liu et al. in 2017 reported that exposure to PM_2.5_ during pregnancy represents an important risk factor for PTB [20].

##### Polycyclic Aromatic Hydrocarbons (PAHS)

PAHs represent a class of substances that are released during combustion processes from vehicles and industries. The exposure may occur through inhalation. Some authors observed a correlation between PAHs exposure and PTB [21,22]. Furthermore, Guo et al. in 2012 confirmed these association, finding a high PAH concentration in umbilical cord blood at birth of newborns as a marker of maternal exposure [23]. Nevertheless, further research is necessary to find more sensitive serum or urinary biomarkers of PAHs exposure that can express the degree of inhalation and ingestion [24,25,26].

#### 3.1.2. Organic Pollutants

##### Drinking Water Contaminants

Many chemicals can be found in drinking water supplies. The treatment of drinking water with chlorine leads to the release of disinfection by-products (DBP), such as trihalomethanes (THM: chloroform, bromoform, bromodichloromethane, and dichlorobromomethane) and haloacetic acids (HAA: chloroacetic acid, dichloroacetic acid, trichloroacetic acid, bromoacetic acid, and dibromo acetic acid). The relationship between the exposure to these contaminants and PTB is not clear. Grellier et al. in 2010 published a meta-analysis of nine studies, showing no significant association with PTB [27,28,29,30,31,32,33,34] and, on the contrary, a possible a reduction in PTB after THM exposure [35]. Similarly, two of the nine studies reviewed did not find a significant correlation between the exposure to HAA and PTB [29,33]. In contrast, in line with other studies, an association between small-for gestational-age (SGA) fetuses and THM exposure during the third trimester was described [36,37].

The difficulties in the evaluation of these results is confirmed by a meta-analysis conducted by Jaakkola et al., which highlighted, contrary to their initial hypothesis, a reduction in the risk of PTB, probably due to the protective role played by the residual chlorine of chlorinated water in preventing maternal infections during pregnancy [38]. 

The maternal urinary levels of trichloroacetic acid (TCAA) during pregnancy was proposed as a biomarker of chlorinated water exposure. Furthermore, TCAA urinary levels seem to be a marker of long-term ingestion of contaminated water [39]. A study conducted using this biomarker did not show any significant correlation between maternal urinary levels and PTB [40].

On the other hand, Horton and colleagues (2011) observed an association between DBP exposure and PTB. In their study THM, HAA, and total organic halide (TOX) were measured in drinking water, finding a significantly higher incidence of PTB only in mothers with occasional and continuous TOX exposure [41].

In addition to DBP, chlorinated solvents such as trichloroethylene (TCE) and tetrachloroethylene (PCE) may be found in drinking water supplies. These solvents can dissolve in water and come into contact with pregnant women through inhalation of vapors or ingestion. Other authors found no correlation between exposure to these compounds and PTB [42,43,44].

Rinsky et al. described an association between atrazine exposure and PTB [45]. Atrazine (6-chloro N-ethyl-N-(1-methylethyl)-1,3,5-triazine-2,4-diamine), is an herbicide used worldwide to control the spread of infesting broadleaf and grassy weeds that interfere with the growth of corn, sugar canes, and other crops. This compound can be present in drinking water and can interfere with endocrine homeostasis, acting as endocrine disruptor. This study found a higher incidence of PTB in mothers highly exposed during pregnancy compared to controls [45].

##### Persistent Organic Pollutants: Organochlorine Compounds (OCPs) and Perfluoroalkylated Substances (PFAS)

OCPs are ubiquitous toxic chemicals, widely used in industry, agriculture, and food. They are pesticides, industrial chemicals or by-product of industrial processes or generated by combustion of organic chemicals. Exposure to them seems to be associated with several reproductive disorders and gynecological diseases such as endometriosis [46,47,48,49,50]. 

These substances bioaccumulate in lipid-rich tissues because of their strong lipophilic nature and low degradation rates. OCPs have been found in different human tissues such as blood, placental tissue, amniotic fluid, and breast milk. [51]. Exposed mothers could transfer these chemicals to the fetus and the newborn through placenta, blood, and breast milk. This transfer was confirmed by several studies [52,53]. Porpora et al. found a strong correlation between maternal and fetal compartments levels of some polychlorinated biphenyls (PCB 153 and 180), β-hexachlorobenzene (β HCB), and *p,p’*-dichlorodiphenyldichloroethylene (*p,p*’-DDE), a metabolite of Dichlorodiphenyltrichloroethane (DDT ) [54].

In other studies, higher levels of βHCB were found in maternal and cord blood in cases of PTB with intact membrane compared to women with full-term babies [55]. The continuous exposure to these compounds during pregnancy can alter hormonal homeostasis: physiologically, progesterone promotes uterine quiescence; estrogens, on the other hand, promote myometrial activation with increased reactivity to uterotonic agents.

OCPs may act as endocrine chemicals disruptor (ECDs) modulating the release, transport, metabolism, or elimination of natural hormones. In mammalians and fishes, β-HCH, the most dangerous of Hexachlorocyclohexane (HCH) isomers, acts as a xenoestrogen, activating uterine contraction with a high risk of PTB [56,57,58].

Moreover, Tyagi et al. demonstrated that these compounds increase the mRNA expression of genes involved in inflammatory pathways and prostaglandins, enhancing uterine contractions and cervical modifications [59]. Furthermore, some authors have shown a relationship between organochlorine and organophosphate pesticides and neurodevelopmental disorders in infants, which was associated with a higher incidence of sudden infant death syndrome (SIDS) [60]. Perfluoroalkyl substances (PFAS) are a family of synthetic compounds composed of a carbon-fluorine backbon. Many PFAS are resistant to oil and water and therefore useful in the manufacture of stain-resistant products (e.g., carpets and fabrics), non-stick coatings, and food packaging [61].

A study of De Felip et al. showed higher serum concentrations of perfluorooctane sulfonate (PFOS) and perfluorooctanoic acid (PFOA) in women with great consumption of beef, pork, lamb, ham and salami, fish and seafood. Higher levels of PFOA were also found in women with a diet rich in cheese, eggs, liver, and vegetables [62]. Sagiv et al. found over two-fold odds of preterm birth in women with concentrations in the highest quartile of PFOS versus those with concentrations in the lowest quartile (odds ratio = 2.4, 95% CI: 1.3, 4.4). Odds of preterm birth were weaker for perfuorononanoate (PFNA) and null for PFOA and perfluorohexane sulfonate (PFHxS) [61].

##### Non-Persistent Organic Pollutants: Phthalates, Phenols, and Parabens

Phthalates are non-persistent organic pollutants used to increase flexibility in plastics and are also found in many personal care products and toys. The exposure to these substances is spread through ingestion, dermal application, and inhalation. They may act as endocrine disruptors [63]. Adibi et al. in 2009 found that i-(2-ethylhexyl) phthalate (DEHP) may interfere with signaling related to the timing of labor. This compound can cross the placenta, disrupt steroid hormone synthesis, and activate peroxisome proliferator-activated receptor gamma. However, this study found that DEHP may have a protective role in PTB occurrence [64]. A Mexican cohort study who compared third-trimester urinary phthalate metabolite concentrations in 30 women who had PTB (<37 weeks of gestation) with those of 30 controls (≥37 weeks of gestation) found that phthalate exposure can be associated with PTB [65]. Ferguson et al., in a case–control study of 130 cases of PTB and 352 controls, found that women exposed to phthalates early and late in pregnancy had significantly increased risk of PTB [24,25]. Few years later, the same group conducted other studies on the correlation between exposure to phthalates, analyzing urinary 8-isoprostane, a marker of oxidative stress, confirming that PTB was due to the inflammatory effects of these compounds [66,67].

Exposition to phenols and parabens is extremely common as a consequence of their high distribution in the environment. They are present in food and personal care products, as well as in many pharmaceutical compounds. Some of the phenols and parabens more frequently present are bisphenol S (BPS), benzophenone 3 (BP3), triclosan (TCS), triclocarban (TCB), methyl-paraben (MPB), ethyl-paraben (EPB), propyl-paraben (PPB), and butyl-paraben (BPB). The role of these toxic compounds in adverse pregnancy outcome is controversial, as they can have a double effect on the immune system, i.e., a proinflammatory or an anti-inflammatory effect. Aung et al., in 2018, reported that urinary levels of TCS, 2,5-dichlorophenol (2-5-DCP), and MPB in mothers’ urine is associated with an increase of IL-6, IL-12, and TNF alpha, probably through the stimulation of the estrogen receptor, while higher urinary concentrations of EPB and BP3 are associated with lower concentrations of proinflammatory cytokines [68].

#### 3.1.3. Tobacco Smoke and Electronic Cigarettes Aerosol

Many studies have observed an association between tobacco smoke and PTB [69,70,71,72]. Despite the decreased prevalence over the years of pregnant women smokers, it is estimated that in the future about 13% of women will still be smoking during pregnancy [73]. Smoking represents one of the few risk factors that can be controlled; therefore, exposure to it can be reduced during pregnancy. The law enforcement forbidding smoking in public places contributed to reduce the exposure in the last few years. However, there are still conflicting data about the relationship between PTB and passive exposure to tobacco smoke.

Cigarettes contain a series of substances, in addition to nicotine, which can have toxic effects, such as CO, cyanide, aniline, methanol, hydrogen sulfide, arsenic, lead, cadmium, and other toxins or carcinogens [74]. These toxins may induce pregnancy disorders through different mechanisms, described by Goldstein et al. [75]. Smoking causes endothelial damage mediated by oxidative stress, which can determine vasoconstriction with fetal growth restriction. Moreover, the increase of CO, with its greater binding affinity for hemoglobin, reduces oxygen diffusion to the fetus [75]. Regarding PTB, smoking may increase the risk of Preterm Premature Rupture of Membranes (PPROM) [5]. Furthermore, smoking reduces the elasticity of the fetal membranes, with higher risk of their rupture and PTB [76].

A meta-analysis conducted by Cui H. et al. in 2016, which included 24 studies involving 5607 women who experienced PTB, found that environmental tobacco passive exposure increases the risk of PTB between 20% and 16% in the external and domestic environment, respectively [77].

Cigarette smoking during pregnancy is also associated with adverse neonatal outcomes, with a higher incidence of SIDS [78,79]. A preponderant role in this sense is played by fetal exposure to nicotine during pregnancy. Evidence suggests that acetylcholine (ACh) has a trophic role in brain development through a mechanism mediated by ACh receptors. It seems that nicotine can bind these receptors, causing their abnormal function and expression, with a consequent neuronal damage [60].

The evaluation of tobacco-smoking consequences during pregnancy can be problematic because of the difficulty in establishing the level of exposure. Many authors proposed different markers such as cotinine, a metabolite of nicotine [80], that can be measured in serum [81] and in neonatal meconium and urine [82]; thiocyanate, a metabolite of hydrogen cyanide, rarely used [83], and exhaled carbon monoxide (CO) levels, measurable by a non-invasive and therefore preferable method [84].

In 2017, Chen et al. conducted a study about the effect of maternal exposure to electronic cigarettes’ compounds during pregnancy using mice models. The use of electronic cigarettes is now widespread, and their diffusion has been facilitated by the intense advertising campaign to which they have been subjected. It is common opinion that these cigarettes are harmless, and their use seems to be frequent among pregnant women, with a percentage of around 15% [85]. In addition to nicotine, which may be present in these devices, the presence of other toxic compounds in the aerosol of these cigarettes has been demonstrated. The aim of Chen’s study was to evaluate the effects of maternal exposure to these aerosols on the fetus, with attention on the possible increase in the expression of the pro-inflammatory cytokines and the development of the respiratory apparatus in the offspring. They found that mice exposed to intrauterine e-cigarettes had an increase in pro-inflammatory cytokines production, independently of nicotine presence in the device, condemning any type of electronic cigarette. They noticed the involvement of three important markers for lung development: PDGFa, EphB2, and Sftpc. PDGFa was increased in both e-cigarette exposure groups, with and without nicotine. Moreover, an abnormal production of pro-inflammatory cytokines in the lung of mothers and offspring, including IL-1ß, IL-6, and TNFa, has been demonstrated. Finally, abnormalities in DNA methylation were also found, even if it is not clear which parts of the genome are involved [86]. At the best of our knowledge, there are not studies about e-cigarettes exposure and the occurrence of PTB.

#### 3.1.4. Toxic Metals

The reproductive and clinical implications of metal exposures are well described by Singh et al. [87]. They observed that the exposure to these substances could have severe consequences on the maternal–fetal unit as well as on the reproductive system. These metals could be involved in the unregulated production of free radicals such as reactive oxygen species (ROS) and reactive nitrogen species (RNS) in endothelial cells. As a result, these interactions could cause damage to DNA, membrane lipids, and enzymes in placental tissues [87].

Moreover, toxic metals could modify the levels of antioxidant biomarkers, such as glutathione (GSH), superoxide dismutase (SOD), and catalase, leading to poor pregnancy outcomes, such as fetal growth restriction, pre-eclampsia, and PTB [88] (Table 1).

## 4. Mechanisms Involved

The placental transfer of pollutants could compromise placental function and pregnancy outcomes directly or indirectly through a wide range of mechanisms:

### 4.1. DNA Damage

Chemical compounds could bind DNA and form covalent adducts, modifying genic expression and fetal development [89,90,91]. Studies on humans have shown that intrauterine exposure to air pollution is associated with increased levels of DNA adducts in the placenta and in fetal blood [92,93].

### 4.2. Hypoxia

Low oxygenation can affect intervillous perfusion and alter maternal blood viscosity [94]. This mechanism is well described in several studies: some air pollutants could cause placental insufficiency and fetal hypoxia diagnosed by an increased value of Lactate dehydrogenase (LDH), an indirect sign of anaerobic metabolism [95,96,97]. Fetal hypoxia could cause the inhibition of breathing movements that can be observed by ultrasound, reduction of the numbers of alveoli, and high airway resistance [98,99].

### 4.3. Oxidative Stress

Oxidative stress is caused by an imbalance between the production of ROS and the ability of antioxidant defenses to neutralize them. It could lead to negative pregnancy and fetal outcomes [100,101,102]. This balance is well controlled by nitric oxide that, through the action of SOD, can inactivate superoxide anions. Inflammation could produce superoxide and nitric oxide, and these two species react to form peroxynitrite; the latter compound is involved in lipid peroxidation and DNA damage, and its toxic activity is shown by the presence of nitrotyrosine residues. In several gestational diseases, high levels of oxidative species are confirmed by the presence of these compounds in placental tissues. These substances could affect placental function, in particular trophoblast proliferation and differentiation, increasing vascular reactivity [103], with poor fetal outcomes. Many environmental compounds can cause cellular oxidative stress, with consequent alteration of the integrity of the amniochorial membrane and/or activation of cervical ripening, a prelude to PTB occurrence [104,105,106,107].

### 4.4. Inflammation

Toxic compounds could also activate inflammatory pathways, with severe consequences for pregnancy [108,109] and fetal development disorders [110]. Nachman et al. 2016 found a positive relationship between maternal PM_2.5_ exposure before and during pregnancy and intrauterine inflammation [111]. Furthermore, it has been demonstrated that PM_2.5_ exposure in rats during pregnancy may induce inflammation in the placenta [112]. Moreover, PM_2.5_ may be associated with intrauterine inflammation (IUI), one of the most relevant risk factors for PTB [113,114,115].

### 4.5. Epigenetic Changes

Environmental pollutants could change genic expression through epigenetic changes (post-translational modifications) such as DNA methylation and acetylation and ubiquitination and histone modifications [116,117,118]. Several studies have shown that maternal toxic exposure correlates to a reduction of placental methylation [119,120]. Air pollution exposure could lead to epigenetic alterations of genes related to the inflammatory pathway [121].

## 5. Not Only Environmental Toxic Compounds: The Role of Global Warming

In the last years, there has been an increasing interest in global warming, both in terms of the impact on the environment and in terms of the impact on human health. Many authors conducted studies about the correlation between global warming and the occurrence of unfavorable pregnancy outcomes, like PTB [122,123,124,125]. 

Zhong et al., in 2018, performed a cohort study to determine the critical period of heat exposure during pregnancy in the occurrence of PTB. The authors found an increased risk of PTB proportional to the time of exposition especially in warm seasons, with a major risk with exposure during the second trimester of pregnancy [126]. Conversely, another review of the literature reported that both heat and cold are associated to higher risk of PTB [127].

As of the window of exposure, a recent study across 12 U.S.A. cities reported a major effect for heat and cold especially during the first seven weeks of gestation for cold and during weeks 15–21 for heat [123].

In addition to these findings, Guo et al. demonstrated a critical window also during the pre-gestational period. Moreover, they found that exposure to cold temperatures in the last third trimester before pregnancy has a protective role. The underlying mechanism of this association remains unclear. It has been assumed that heat exposure may lead to acute stress, with blood flow reduction to the uterus and onset of contractions due to a massive release of oxytocin [128].

## 6. Discussion

Over the last few years, we have witnessed an increase in the incidence of PTB. The PTB syndrome represents a worldwide problem, and the comprehension of the underlying mechanisms is necessary in order to prevent it, when possible, and to offer to mother and fetus a better treatment to reduce the related complications. In order to develop the PTB syndrome, three factors are necessary: a valid uterine contractile activity, cervical ripening, and amniotic membranes activation followed by their preterm rupture (PPROM). The cause of PTB is not always clear. The most commonly described causes of PTB are related to infectious and inflammatory factors. Inflammation and the activation of inflammatory cytokines play a fundamental role in PTB, and it seems that exposure to chemical compounds and pollutants may be associated with this obstetric complication precisely through the activation of inflammatory cytokines. Available data on the role of environmental pollutants in the occurrence of PTB provide conflicting results. Data cannot be well compared because of different study designs, analytic methods, and geographical areas where the studies were carried out. 

Therefore, the relationship between environmental pollutants and PTB remains unclear. However, there are some mechanisms that may explain the role of these substances as risk factors for PTB, such as competition with hemoglobin binding sites reducing O2 availability by air pollutants [17]—with consequent fetal growth restriction and fetal distress, which can lead to the activation of labor via the cortisol axis—capability of PM to affect the hemodynamic system through different pathways [129], placental damage, with unregulated production of free radicals such as ROS and RNS in the placental tissue due to the presence of toxic metals [88].

OCPs seem to be able to alter hormonal homeostasis acting as endocrine disruptors. The involvement of the estrogen receptor, of the immune system, and consequently of inflammatory factors are also implicated as the mechanisms of action of phenols and parabens [68]. Despite some POPs are either banned or restricted under the “Stockholm Convention on POPs’ of 2001, entered into force on 17 May 2004, they continue to be present all over the world. In fact, in some countries, these substances are still produced, causing a worldwide exposure which occurs mainly through the food chain. Among all European countries, Italy seems to be the country with the lowest exposure rate, probably due to a greater use of local products [130]. 

Conversely, despite the initial hypotheses, a significant correlation between preterm birth syndrome and drinking water contaminants such as the by-products DBP, THM, and HAA has not been demonstrated, while an association has been described with chlorinated solvents and atrazine, acting on hormone receptors [35,41,45].

It is worth noting that a different role in causing PTB is played by global warming. Particularly, it seems that higher temperatures can lead to oxytocin’s release and onset of uterine contractions. The underlying mechanisms are still unclear. In preventing global warming, an important role is played by green spaces and vegetation. Vegetation influences ambient temperature through different mechanisms as the photosynthesis process, in which part of solar radiation is absorbed, and only a small part is stored as heat. Also, vegetation can modify air movement. For these reasons, a recommended approach could be to increase green areas near urban areas to induce cooling [131].

In conclusion, pollutants seem to influence the occurrence of PTB (Table 2); however, it is very difficult to identify and quantify their role because of conflicting results in the available studies. Nevertheless, being the relationship between PTB syndrome and environmental pollutants an important issue, further studies are sorely needed to standardize exposure analytic methods and to improve comparability.

## Figures and Tables

**Table 1:** Possible effects of exposure to toxic metals during pregnancy.

Toxic Metal	Limit Values in Drinking Water (WHO)	Source of Exposition	Risks of Exposition During Pregnancy
Lead (Pb)	0.05 mg/L	Water, food, air, soli, dust	Passive diffusion to placental tissue Oxidative stress Placental tissue injury High rate of preterm delivery
Cadmium (Cd)	0.003 mcg/L	Fiber-rich foods Vegetables Cereals Potatoes Spinach Air pollution Tobacco smoking	Toxic effect on villous cells with risks of IUGR (intrauterine growth restriction) Preterm delivery Placental hemorrhage Hormonal unbalance Genotoxicity Fetotoxicity
Mercury (Hg)	1 mcg/L	Food (fish) Cosmetic preservatives Insecticides	High binding on placental tissue leads to: Oxidative stress SGA (small for gestational age)
Arsenic	10 mcg/L	Home tiles Industry Agriculture	Anomalous placental vascularization and oxidative stress: Miscarriage Stillbirth Preterm birth Neonatal death

**Table 2:** Studies on environmental chemical exposure and preterm birth. PCB, polychlorinated biphenyls; PPROM, Preterm Premature Rupture of Membranes.

Class of Toxic	Chemicals Compounds	References	Findings
Air pollutants	CO, NO, NO_2_, SO_2_, O_3_	Parker JD 2011 [13], Shah PS 2011 [14], Estarlich M 2015 [15], Fleisher NL 2014 [16], Pike K 2012 [17]	Significant association with adverse gestational outcomes
Particulate matters	Toxics with aerodynamic diameter (PM_10_-PM_2.5_)	Jedrychowski WA 2012 [19], Liu C 2017 [20]	Positive correlation with risk of PTB
Polycyclic aromatic hydrocarbons (PAHS)	Coal or fossil fuel, forest fires, waste incineration,	Dong, X. 2018 [26]; Ferguson KK JAMA pediatrics 2014 [24]; Padula AM 2014 [21]; Wilhelm M 2011 [22]; Guo Y 2012 [23]	PAHs concentration in umbilical cord blood correlates with negative pregnancy outcomes
Drinking water contaminants	Disinfection by-products, atrazine	Grellier J 2010 [35], Hoffman CS 2008 [29]; Wright JM 2004 [33], Graves CG 2001 [36], Tardiff RG 2006 [37], Jaakkola JJ 2001 [38], Costet N 2012 [40], Horton BJ 2011 [41], Bove FJ 1995 [42]; Aschengrau A 2008 [43]; Forand SP 2011 [44], Rinsky JL 2012 [45]	Unclear correlation with risk of PTB
Persistent organic compounds	Organochlorine Compounds Perfluoroalkylaed substances	Porpora MG 2013 [54], Pathak R 2008 [55], Wood SL 2007 [56], Tyagi V 2016 [59] Sagiv SK 2018 [61]	High concentration of PCB in maternal and fetal compartments correlates with negative pregnancy outcomes
Not persistent organic compounds	Phthalates, Phenols, and Parabens	Adibi JJ 2009 [64], Meeker JD 2009 [65], Ferguson KK 2014 [24], Ferguson KK 2014 [25]	Unclear correlation with risk of PTB
Tobacco smoke and e-cigarettes	Nicotine, CO, cyanide, aniline, methanol, hydrogen sulfide, arsenic, lead, cadmium.	Goldenberg RL 2008 [5], Lavezzi AM 2015 [60], Crane J 2011 [69]; Qiu J 2014 [70]; Salmasi G 2010 [71]; Ion RC 2015 [72], Cnattingius S 2004 [73], Banowitz NL 2000 [74], Goldstein H 1964 [75], Hadley CB 1990 [76], Cui H 2016 [77], Miller RL 2004 [78], Behrooz L 2018 [79], Benowitz NL 1996 [80], Shipton D 2009 [81], Ostrea EM 1994 [82], Stevens KR 2004 [83], Secker-Walker RH 1997 [84], Wagner NJ 2017 [85], Chen H 2018 [86].	Increasing risk of PPROM, oxidative stress, fetal growth restriction
Toxic Metals	Lead, cadmium, mercury, arsenic	Singh L 2018 [87], Chen Z 2001 [88]	Positive association with risk of PTB
Global warming		Arroyo V. 2016 [122], Ha S. 2015 [123], Kloog I. 2015 [124], Avalos LA. 2017 [125], Zhong Q. 2018 [126], Kloog I. 2019 [127], Gou T. 2018 [128]	Positive correlation with PTB
